# How stem cell composition in bone marrow aspirate relates to clinical outcomes when used for cervical spine fusion

**DOI:** 10.1371/journal.pone.0203714

**Published:** 2018-09-24

**Authors:** Christopher D. Chaput, Adam Shar, Daniel Jupiter, Zach Hubert, Bret Clough, Ulf Krause, Carl A. Gregory

**Affiliations:** 1 Department of Orthopedics, University of Texas Health San Antonio, San Antonio, Texas, United States of America; 2 Medical Education Building, Texas A&M Health Science Center, Temple Campus, Temple, Texas, United States of America; 3 Department of Preventive Medicine and Community Health, The University of Texas Medical Branch, Galveston, Texas, United States of America; 4 Institute for Regenerative Medicine, Texas A&M Health Science Center, College Station, Texas, United States of America; 5 Institute for Transfusion Medicine and Transplant Immunology, University Hospital Muenster, Muenster, Germany; Centro Cardiologico Monzino, ITALY

## Abstract

Anterior cervical discectomy and fusion (ACDF) is performed to relieve pain caused by degenerative disk disease and nerve obstruction. As an alternative to bone graft, autologous concentrated bone marrow aspirate (CBMA) is used to achieve vertebral fusion with a satisfactory success rate. This has been attributed in part to bone marrow-resident mesenchymal stromal cells (MSCs) with the capacity to differentiate into osteoblasts and generate bone tissue. To date, there has been no study comparing cellular yields, MSC frequencies and their osteogenic potential with ACDF outcome. Patients (n = 24) received ACDF with CBMA and allograft bone matrix. Colony forming unit fibroblast (CFU-F) and CFU-osteoblasts (CFU-O) assays were performed on CBMA samples to enumerate MSCs (CFU-F) and osteogenic MSCs (CFU-O). CFUs were normalized to CBMA volume to define yield and also to mononuclear cells (MNC) to define frequency. After 1-year, fusion rates were good (86.7%) with pain and disability improved. There was a negative relationship between MNC and CFU-F measurements with age of patient and CFU-Os negatively correlated with age in females but not males. Tobacco use did not affect CBMA but was associated with poorer clinical outcome. Surprisingly, we found that while high-grade fusion was not associated with CFU-O, it correlated strongly (p<0.0067) with CBMA containing the lowest frequencies of CFU-F (3.0x10^-6^–5.83x10^-5^ CFU-F/MNC). MNC levels alone were not responsible for the results. These observations suggest that osteogenesis by human bone marrow is controlled by homeostatic ratio of MSCs to other cellular bone marrow components rather than absolute level of osteogenic MSCs, and that a lower ratio of MSCs to other cellular components in marrow tends to predict effective osteogenesis during ACDF. The results presented herein challenge the current dogma surrounding the proposed mechanism of MSCs in bone healing.

## Introduction

Anterior cervical discectomy and fusion (ACDF) remains the mainstay of surgical treatment for patients with nerve compression due to narrowing of the vertebral channels that harbor the nerves of the spinal column caused by excessive bone growth and intervertebral disk herniation [1]. Depending on the pathology, the decompression process consists of removal of intervertebral disc(s), partial or complete removal of the offending vertebral body and removal of osteophytes. The resulting void is then filled to restore the physiological height of the vertebral column using a variety of materials, such as iliac crest autograft, cadaveric bone allograft, synthetic scaffolds such as hydroxyapatite and tricalcium phosphate, and interbody cages [2]. This is frequently supplemented by synthetic plating to aid in supporting the construct during the bony fusion process. Iliac crest bone graft is considered the “*gold standard*” for bone grafting in cervical fusion due to its osteoconductive and osteoinductive properties as well as its osteogenic potential [3]. However, its drawbacks include donor site morbidity, limited graft volume, and limited re-harvesting capability. Alternatively, bone marrow aspirate (BMA) obtained from the iliac crest also has osteoinductive and osteogenic properties and can be used in conjunction with an osteoconductive agent, such as an allograft or a synthetic scaffold [4]. Compared to iliac crest bone grafting, the use of BMA is less invasive and is associated with lower harvest site morbidity [5]. Furthermore, it is a source of mesenchymal stem cells (MSCs), presumptive progenitors of osteoblasts and a potent source immune-regulatory, angiogenic and anti-apoptotic trophic factors [6] as well as a variety of other cell types responsible for osteogenic and angiogenic processes [7]. As such, multiple studies have shown that BMA used in combination with a synthetic scaffold in spinal fusion surgeries have high fusion rates [8–11].

BMA can be processed to concentrated bone marrow aspirate (CBMA), which results in a product with a higher concentration of nucleated bone marrow cells compared to bone marrow aspirate (BMA). One of the drawbacks of BMA and CBMA is that host factor variability affects the overall yield of nucleated cells and thus, the number of putative osteoprogenitor cells available within the sample [12, 13]. For repair of non-union injuries of long bones, the number of putative osteoprogenitor cells, as defined by the plastic adherent, fibroblastoid, mesenchymal component of the bone marrow, has been shown to correlate with efficacy [13]. However, no study to date has evaluated the relationship between the cellular characteristics of CBMA utilized in ACDF with radiographic evidence of fusion and clinical outcome.

The purpose of this study is to identify how CBMA cell yield, CBMA-resident MSCs defined as fibroblastoid colony forming units (CFU-F), osteogenic CFU-F cells (CFU-O), donor age, gender and tobacco usage might affect ACDF in terms of fusion outcome and reduction of pain and disability.

## Materials and methods

### Clinical procedures

This study was reviewed and approved by the Baylor Scott & White Institutional Review Board. IRB approval number 081934. Participants provided written informed consent. Consent forms are archived at the Baylor Scott and White Department of Orthopedics. From December 2009 to May 2012, we prospectively collected data from 24 consecutive and consenting patients who underwent one-level or contiguous 2- or 3-level ACDF performed by a single surgeon (CDC) using the Smith-Robinson approach for the treatment of degenerative disc disease, degenerative cervical spondylolisthesis, spinal stenosis, cervical radiculopathy, or cervical myelopathy [14]. Exclusion criteria consisted of patients with history of previous instrumentation and fusion at the intended intervention level, patients with history of bilateral iliac crest bone graft harvest, patients with active infection (either systemic or localized to implantation/aspiration site), and patients with less than 1 year of follow-up. Eligible patients were also required to complete a pre-operative Neck Disability Index (NDI), a questionnaire-based measure of pain and disability [15]. In order to obtain CBMA, 60 mL of BMA were collected from the iliac crest using a 5-hole aspirator. A small BMA sample was retained (2–5 mL) and the remaining BMA was processed through a SmartPrep 2 System (Harvest Technologies Corp, Plymouth, MA) to produce CBMA. An aliquot of CBMA (2–5 mL) was retained for cell analysis, while the remaining CBMA was combined with cancellous allograft bone and that mixture was placed within and around a poly-ether-ether-ketone (PEEK) cage. The PEEK cage was then implanted into the area of void. All fusions were supplemented with anterior cervical instrumentation. All patient identifying data were securely maintained at Baylor Scott & White hospital. With the exception of clinical staff directly involved with patient care, investigators were blinded to the identity of patients involved in the study.

### Clinical outcome measures

**Fig 1:**
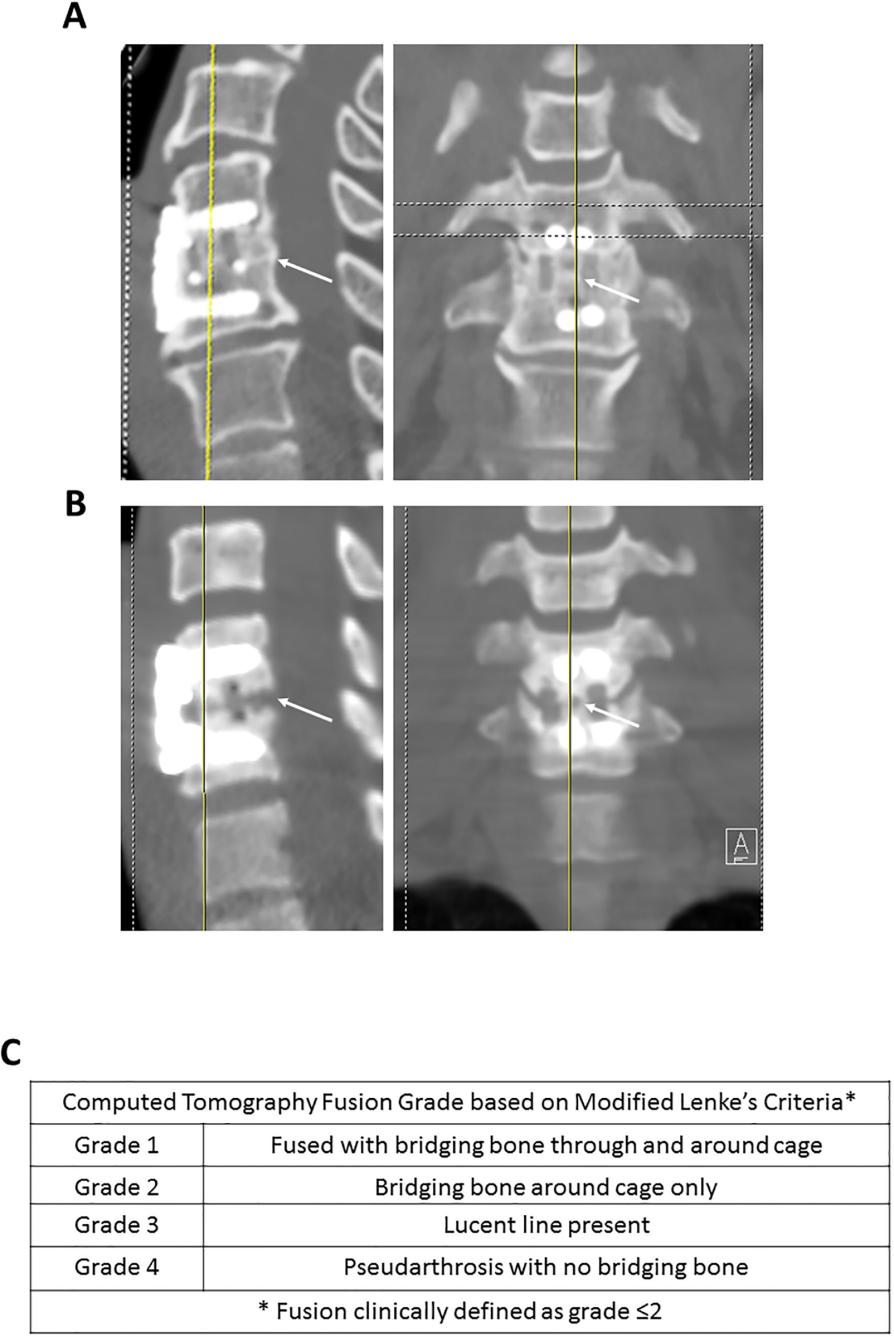
Modified Lenke’s criteria for defining fusion. (A&B): Examples of saggital (*left*) and coronal (*right*) CT reconstructions demonstrating *Grade 1*, defined as solid fusion with bridging bone through and around cage in the interbody space (*arrowed*, A), and *Grade 3* with radiolucent line within the interbody space and likely pseudoarthrosis (*arrowed*, B). (C) Summary of definitions.

Upon recruitment to the study, gender, age, tobacco use and NDI, was recorded. At the 1-year post-operative visit, 14 (58%) patients completed a 10-point hip visual analog score (VAS) to evaluate donor site pain (0 = no pain, 10 = worst possible pain) and also completed a second NDI to quantify changes between pre-operative and post-operative neck disability. At the 1-year post-operative period they also underwent computed tomographic scanning (CT) of the cervical spine (Fig 1A and 1B). Grading of radiographic fusion, was performed using the modified Lenke’s criteria, with successful fusion being defined as grade 1 or 2 (Fig 1C) [16]. The CT images were evaluated by a trained, independent observer.

### Processing of bone marrow

**Fig 2:**
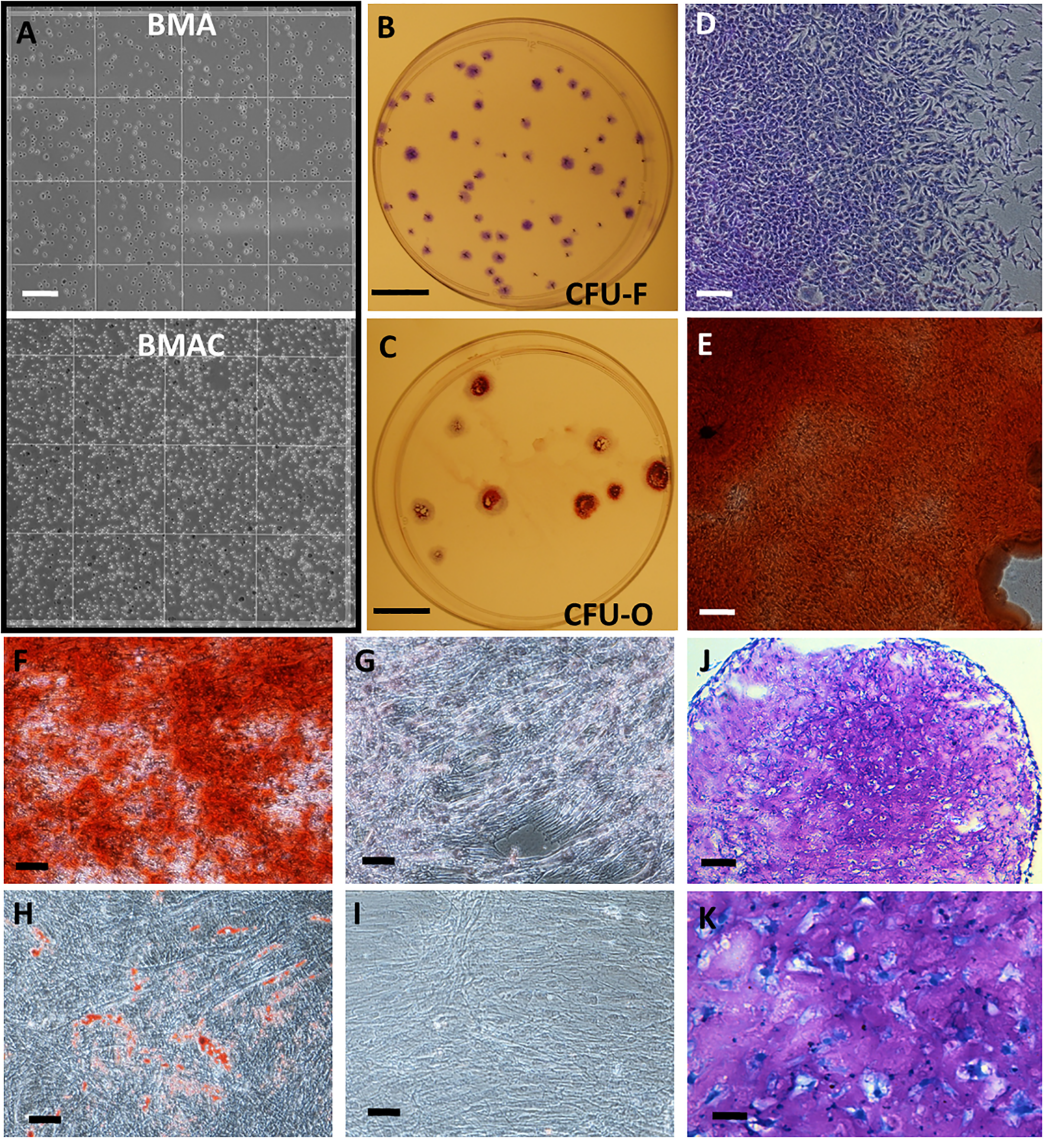
Morphology of CBMA and CFUs and a representative MSC preparation from one of the CBMA specimens demonstrating differentiation into mineralizing osteoblasts, adipocytes and chondrocytes. **(A)** Micrograph of hemacytometer containing unconcentrated BMA (*left*) and the same sample after concentration (*right*). Tryphan blue stained cells represent <5% of the population. Scale bar = 0.1 mm **(B)** Typical appearance of crystal violet-stained CFU-F plate. Scale bar = 30 mm. (**C)** Typical appearance of alizarin red S-stained CFU-O plate. Scale bar = 30 mm. **(D):** Typical appearance of crystal violet-stained CFU-F colony. Scale bar = 250 μm. **(E)** Typical appearance of alizarin red S-stained CFU-O colony. Scale bar = 250 μm. **(F)** Osteogenic monolayer stained with alizarin red S to indicate calcified matrix. Scale bar = 250 μm. **(H)** Adipogenic monolayer stained with oil red O to visualize internal lipid droplets. Scale bar = 250 μm. **(G&I)** Control (undifferentiated) monolayer stained with alizarin red S and oil red O respectively. Scale bar = 250 μm. **(J&K)** Chondrogenic micro-mass pellet, sectioned and stained with toluidine blue to visualize sulphated proteoglycans and chondrocyte lacunae. Scale bar = 200 μm (A), 50 μm (B).

CBMA and BMA samples (2–5 mL) were processed via a Ficoll gradient to recover the mononuclear cells (MNCs). The number of MNCs and their viability was determined by hemacytometer counts and trypan blue exclusion (Sigma Aldrich, St Louis, MO). In all cases, viability was above 95% at the time of culture (Fig 2A).

### CFU assays

To obtain the measurements of plastic-adherent single-cell derived colony forming units (colony forming unit fibroblasts: CFU-F) in CBMA samples, 1x10^6^ viable MNCs were plated in a 154 cm^2^ tissue culture dish (Corning Lifesciences, Tewksbury, MA) in the presence of 25 mL complete culture media (CCM) consisting of alpha-minimal essential medium (α-MEM), 2 mM additional glutamine, 100 U/mL penicillin, 100 μg/mL streptomycin and 20% (v/v) non-heat inactivated fetal bovine serum (Atlanta Biologicals, Norcross, GA). Unless otherwise stated, all tissue culture materials were acquired from Life Technologies (Grand Island, NY). Triplicate plates were cultured in CCM for 3 weeks without media replacement at 37°C in humidified air with 5% (v/v) CO_2_. At 3 weeks, colonies were stained with 2% (w/v) crystal violet dye (Sigma) dissolved in 95% methanol. Colonies >3 mm in diameter were scored and reported as mean CFU-F (n = 3) per mL of original drawn sample (defined herein as *yield*) or CFU-F per MNC (defined herein as *frequency*) (Fig 2B and 2D).

To obtain measurements of osteogenic colony forming units (colony forming unit osteoblasts: CFU-O) in CBMA samples, 1x10^6^ viable MNCs were plated in a 154 cm^2^ tissue culture dish in the presence of CCM for 2 weeks. Thereafter, the media was converted to osteogenic differentiation media consisting of CCM containing 5 mM β-glycerophosphate, 50 μg/mL ascorbic acid and 1 nM dexamethasone (Sigma) and cultured with replacement at 1 week for 2 further weeks. Cultures were then washed in phosphate buffered saline (PBS) fixed in buffered 4% (v/v) paraformaldehyde (Sigma) for 20 min, washed in dH_2_O, and stained for 15 min in alizarin red S (ARS) solution prepared in 10% (v/v) acetic acid adjusted to pH 4.1 with ammonium hydroxide. Plates were washed with copious dH_2_O until background staining was absent. Deep red colonies, containing calcified extracellular matrix, were scored and reported as mean CFU-O (n = 3) per mL of original drawn sample (defined herein as *yield*) or CFU-O per MNC (defined herein as *frequency*) (Fig 2C and 2E).

### Expansion and subculture of MSCs

In some cases, MSC preparations were derived from the specimens. For this purpose, MNCs were seeded in tissue culture treated plates (Nunc Cell Bind, Thermo Fisher Scientific, Waltham, MA) at a density of 1x10^5^ per cm^2^ fed with CCM until fibroblastoid, plastic adherent colonies were visible. Thereafter, plates were washed in phosphate buffered saline (PBS) followed by changes of CCM every 3–5 days for 10–14 days to expand the plastic adherent cells and deplete non-adherent hematopoietic cells. Upon establishment of a 50% monolayer of plastic adherent MSCs, they were recovered with trypsin/EDTA and subcultured for expansion. For expansion, cells were plated at an initial seeding density of 100 cells per cm^2^ and allowed to divide for 6–8 doublings until 40–50% confluent. For re-passage and experiments, MSCs were recovered with trypsin/EDTA. For cryopreservation, cells were suspended in freezing media α-MEM containing 30% (v/v) FBS and 5% (v/v) dimethyl sulphoxide (Hybrimax, Sigma-Aldrich, St Louis, MO).

### Immunophenotyping

MSCs were immunophenotyped using a Cytomics FC500 flow cytometer (Beckman Coulter, Indianapolis, IN). Data were processed using the manufacturers’ software (CXP, Beckman Coulter). Antibodies against the following cell surface antigens were used: CD14 (RMO52), CD19 (J3-119), CD34 (581), CD45 (J.33), CD73 (AD2), CD90 (Thy-1/310), CD105 (IG2), CD146 (TEA1/34), CD166 (3A6), HLA-A, B, C (G46-2.6), HLA-DP, DQ, DR (Tu39).

### Osteogenic differentiation of MSCs

Confluent monolayers of MSCs were incubated in CCM containing 10^−8^ M dexamethasone, 50 μg/mL ascorbic acid and 5 mM β-glycerol phosphate for 21 days with changes every 2 days. The monolayers were then stained with ARS. Micrographs were taken using a Nikon Eclipse, TE200 inverted microscope fitted with a Nikon DXM1200F digital camera.

### Adipogenic differentiation of MSCs

Confluent monolayers of hMSCs were incubated in CCM supplemented with 0.5 μM dexamethasone, 5x10^-8^ M isobutylmethylxanthine, and 5x10^-7^ M indomethacin. Media were changed every 2 days. After 21 days, the cultures were fixed in 10% (v/v) formalin and stained with 0.5% (w/v) oil red-O solution in 30% (v/v) isopropanol and PBS. The dishes were washed with PBS and visualized using an inverted microscope (Nikon Eclipse, TE200).

### Chondrogenic differentiation

Pellet cultures containing 200,000 hMSCs were incubated in high glucose Dulbeco’s-MEM containing 10^−7^ M dexamethasone, 50 μg/mL ascorbate-2-phosphate, 40 μg/mL proline, 100 μg/mL pyruvate and 2x ITS plus premix with changes every 3 days. After 21 days, the pellets were washed in PBS and fixed in 4% (v/v) paraformaldehyde, embedded in paraffin, sectioned, and then stained with toluidine blue to visualize proteoglycans and lacunae.

### Statistics

All data were summarized as means with standard deviation (SD) or standard error of the mean (SEM). For analysis of means and distributions the ranges, 95% confidence intervals (CI) and medians are also calculated. For correlation analysis of cell-based data, all datasets were tested for normality (D’Agnostino & Pearson test) prior to Pearson or Spearman correlation analysis. Datasets were also tested for significant outliers using the method of Grubb or Motulsky and Brown [17]. Binary data were analyzed by Fisher Exact test and pairwise comparisons of continuous data were analyzed by the Student’s t-test. %NDI measurements were compared using the Wilcoxon matched-pairs signed rank test. In all cases, calculations were performed using GraphPad Software (GraphPad Prism version 5.00 for Windows, GraphPad Software, San Diego California USA, www.graphpad.com). In accordance with current convention, statistical significance was arbitrarily designated at p < 0.05 but p-values are reported for all tests.

## Results

### Patient demographics, BM recovery and concentration

Twenty four patients with a total of 31 spinal levels met the inclusion criteria. The average age of the patients in the study was 46.8 years. Fourteen out of twenty four patients were females and 10/24 patients were males and 50% of the patients were tobacco users (Table 1). The SmartPrep-2 System concentrated BMA at an average of 3.94 fold while maintaining viability above 95% (Fig 2A). CFU counts were normalized to the original volume of the CBMA aspirate (hereafter referred to as CFU/mL or *yield*) and also normalized to the number of MNC (hereafter referred to as CFU/MNC or *frequency*). MNC recovery and CFU measurements have the capacity to vary by 2 orders of magnitude between donors (Table 1).

**Table 1:** Demographics, %NDI, Hip VAS and CBMA and CFU data.

**Mean age**	46.8 years (32–67, SD 9.32)
**Gender (n)**	
**Female**	58.3% (14)
**Male**	41.7% (10)
**Tobacco use? (n)**	
**Yes**	50% (12)
**No**	50% (12)
**%NDI**	
**Pre-operative (range, SD, 95% CI, median)**	48.00 (2.00–82.00, 18.76, 40.08–55.92, 48.00)
**Post-operative (range, SD, 95% CI, median)**	21.08 (0.00–70.00, 19.18, 12.98–29.18, 16.00)
**Δ%NDI (range, SD, 95% CI, median)**	-26.92 (-52.00–10.00, 16.88, -34.04- -19.79, -32.00) pre vs post p-value <0.0001
**Mean Hip VAS (range, SD, 95% CI, median)**	1.07 (0–8, 2.369, -0.2961–2.439, 0)
**CBMA mean (range, SD, 95% CI, median)**	
**MNCs/mL**	4.032x10^7^ (3.42x10^6^-1.08x10^8^, SD 2.842x10^7^, CI 2.803x10^-7^–5.216x10^-7^, median 3.358x10^-7^)
**CFU-Fs/mL**	1914 (43–6549, SD 1535, CI 1235–2592, median 1815)
**CFU-Os/mL**	858 (10–2479, SD 761, CI 529–1187, median 657)
**CFU-Fs/MNC**	4.761 x 10^−5^ (3x10^-6^–1.24x10^-4^, SD 2.77x10^-5^, CI 3.56x10^-5^–5.96x10^-5^, median 4.3x10^-5^)
**CFU-Os/MNC**	2.1 x 10^−5^ (6.67x10^-7^–6.3x10^-5^, SD 1.65x10^-5^, 1.366x10^-5^–2.831x10^-5^, median 1.7x10^-5^)

Demographic data, clinical outcome, and MNC and CFU counts after CBMA. %NDI values are based on the Neck Disability Index of Vernon and Mior where 0% refers to no pain or disability and 100% refers to the maximum level of debilitation. Pre and post NDI comparisons performed by Wilcoxon matched pairs signed rank test. VAS refers to a 10 point visual analog pain score where 0 = no pain, 10 = worst possible pain). SD refers to standard deviation, CI refers to 95% confidence interval.

### Analyses on established cultures of MSCs

In 10 cases, established cultures of MSCs were prepared from CBMA samples. Cultures were expanded and tested for commonly accepted criteria for MSC identity [18]. Under standard assay conditions, the MSC preparations differentiated into mineralizing osteoblasts (Fig 2F and 2G), adipocytes (Fig 2H and 2I) and chondrocytes (Fig 2J and 2K). The cells also expressed the appropriate immunophenotype commonly associated with MSCs [18]. Collectively, these data demonstrated that the CBMA procedure did not appear to affect the recovery and behavior of MSCs.

### Effect of donor age on MNC recovery, CFU-F and CFU-O

**Fig 3:**
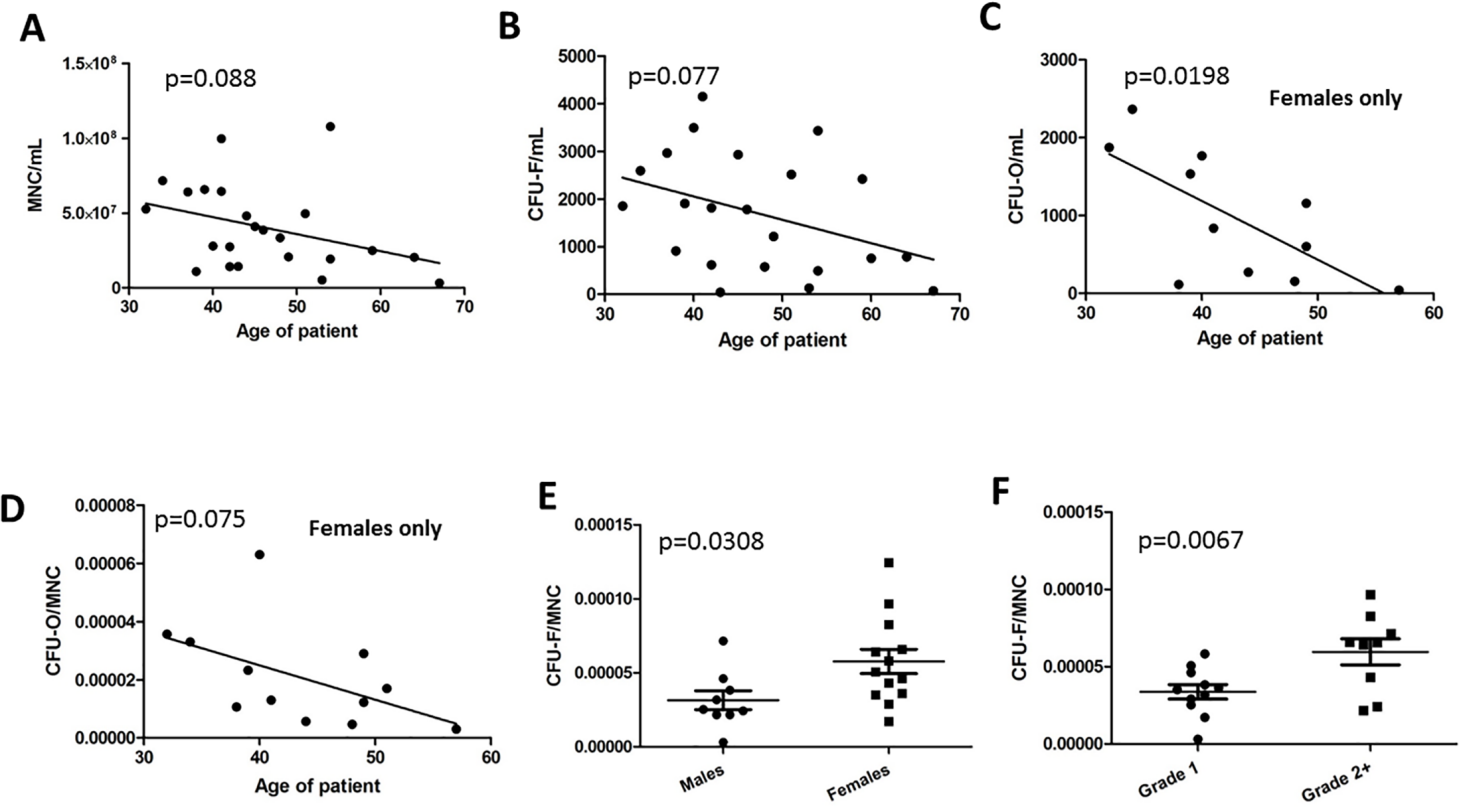
Statistical analyses of cellular parameters, patient demographics and clinical outcome. Analyses by yield (A-C) and by frequency (D-F). (A) Negative trend between MNC recovery and age of patient. (B) Negative correlation between CFU-F/mL (yield) measurements and age of patient. (C) Negative correlation between CFU-O/mL (yield) and age of patient in females. (D) Negative trend between CFU-O/MNC (frequency) and age of patient in females. (E) CFU-F/MNC (frequency) counts are significantly lower in males than females. (F) CFU-F/MNC (frequency) values are significantly lower from patients that exhibit optimal fusions (*Grade 1*, *Grade 1*,*2*, *Grade 1*,*1*) when compared to the remainder of the data set (*Grade 2+*). Correlation analyses were performed using the Pearson test, pairwise comparisons were performed using the Student’s T-test. Error bars represent standard deviations and horizontal lines represent the mean.

Statistical analyses were performed to identify correlations between characteristics of CBMA samples and the age of the donor (Table 2). We found that MNC recovery (normalized to volume of CBMA) and the CFU-F yield (CFU/mL sample), exhibited a slight negative trend with age (Fig 3A and 3B). As expected, when CFU-F values were normalized to the number of MNCs recovered rather than volume of sample (frequency), the relationship was lost because both parameters diminished with age to a roughly equal degree (Table 2). Surprisingly, CFU-O yield and frequency were unaffected by age when genders were combined. When males were analyzed alone, we detected no age-dependent effects on CFU-O frequency or yields, but female samples were subjected to the same tests, CFU-O yields negatively correlated with age (p = 0.0198. Fig 3C) there was also a negative trend with CFU-O frequency (p = 0.075, Fig 3D). These results were not due to an overall reduction in the frequency or yield of CFU-F in female CBMA samples (Table 3). Together, these data indicate that yields of MNCs and resultant CFU-F forming cells therein diminish with age normalized to the volume of the CBMA aspirate, but the frequency of CFU-F in MNC is not affected. Furthermore, age negatively influences overall CFU-O yields and frequencies in female patients but not males.

**Table 2:** Correlation analyses of MNC recovery CFU counts with age.

Test	Sex	Test	P-value	r value (CI)	Notes
**MNCs/mL vs age**	All	Pearson	0.088	-0.36 (-0.67–0.057)	negative trend^ǂ^
**CFU-F/mL vs age**	All	Pearson	0.077	-0.38 (-0.69–0.044)	negative trend^ǂ^
**CFU-O/mL vs age**	All	Pearson	0.71	0.083 (-0.35–0.49)	
**CFU-F/MNC vs age**	All	Pearson	0.30	-0.36 (-0.67–0.058)	
**CFU-O/MNC vs age**	All	Spearman	0.25	-0.28 (-0.66–0.22)	
**CFU-O/mL vs age**	Males	Pearson	0.77	-0.12 (-0.76–0.63)	
**CFU-O/MNC vs age**	Males	Pearson	0.29	0.39 (-0.36–0.84)	
**CFU-F/mL vs age**	Males	Pearson	0.39	-0.32 (-0.81–0.43)	
**CFU-F/MNC vs age**	Males	Pearson	0.26	-0.39 (-0.82–0.31)	
**CFU-O/mL vs age**	Females	Pearson	0.0198	-0.69 (-0.91- -0.15)	negative correlation*
**CFU-O/MNC vs age**	Females	Spearman	0.075	-0.54 (-0.86–0.069)	negative trend^ǂ^
**CFU-F/mL vs age**	Females	Pearson	0.71	-0.11 (-0.63–0.46)	
**CFU-F/MNC vs age**	Females	Pearson	0.41	0.25 (-0.35–0.703)	

Correlation analyses between MNC recovery or CFU frequency with donor age. In the notes column,

^ǂ^ represents a potential trend (p>0.05, <0.1) and

* represents statistical significance. CI represents 95% confidence interval.

**Table 3:** Relationship between gender and tobacco use on CBMA.

Comparison	Mean and SEM	Test	P-value	Notes
**MNC recovery vs gender**	Males 3.893x10^-7^ ± 1.299x10^-7^ Females 4.121x10^-7^ ± 5.544x10^-6^	T-test Welch’s correction	0.87	
**CFU-F/mL vs gender**	Males 1698 ± 658.8 Females 2079 ± 302.8	T-test Welch’s correction	0.57	
**CFU-F/MNC vs gender**	Males 3.154x10^-5^ ± 6.408x10^-6^ Females 5.773x10^-5^ ± 8.230 x10^-6^	T-test	0.03	Higher in females*
**CFU-O/mL vs gender**	Males 726.2 ± 278.8 Females 945.7 ± 193.9	T-test	0.51	
**CFU-O/MNC vs gender**	Males 1.507x10^-5^ ± 4.456x10^-6^ Females 2.472x10^-5^ ± 4.922x10^-6^	T-test	0.19	
**MNC recovery vs tobacco use**	No 3.663x10^-7^ ± 5.831x10^-6^ Yes 4.434x10^-7^ ± 1.085x10^-7^	T-test	0.54	
**CFU-F/mL vs tobacco**	No 2314 ± 565.9 Yes 1477 ± 267.8	T-test Welch’s correction	0.20	
**CFU-F/MNC vs tobacco use**	No 5.094 x10^-5^± 9.349x10^-6^ Yes 4.442x10^-5^± 7.213x10^-6^	T-test	0.59	
**CFU-O/mL vs tobacco use**	No 834.7 ± 224.0 Yes 887.3 ± 234.8	T-test	0.87	
**CFU-O/MNC vs tobacco use**	No 2.022x10^-5^ ± 3.631x10^-6^ Yes 2.173x10^-5^ ± 6.464 x 10^−6^	T-test	0.84	

Effects of gender and tobacco use on CBMA cellularity.

### Effects of gender and tobacco on MNC recovery, CFU-F and CFU-O

We next examined whether gender had an effect on MNC recovery and CFU yield (CFU/mL) or frequency (CFU/MNC) (Table 3). We found that while MNC recovery and CFU yields were unaffected by gender, CFU-F frequency was significantly higher in females than in males (p = 0.0308, Fig 3E) and MNC recoveries or age did not to account for the observation. Tobacco use did not significantly affect the cellular parameters measured.

### CBMA parameters and clinical outcome

The overall average pre-operative %NDI was 48.0% (moderate to severe disability) and after the 1-year post-operative visit it was 21.1% (mild disability), resulting in an overall change in NDI by -26.9% (Table 1). %NDI improved pre-operatively to post-operatively in 21 out of 24 patients, by a mean of -31.4% and worsened pre-operatively to post-operatively in 3 out of 24 patients, by a mean of +4.7%. While we detected no significant statistical relationships between CBMA measurements and change in %NDI, ACDF with CBMA significantly improved overall neck pain and disability parameters (p<0.0001). The hip VAS score at the 1 year post-operative visit was completed in 14 patients and it averaged 1.07, indicating that for the majority of patients, there was little or no pain at the donor site after 1 year (Table 1).

Based on the modified Lenke’s criteria (Fig 1), successful arthrodesis occurred in 26 out of 31 vertebral levels (86.7% fusion rate). Seven of the 24 patients underwent a 2-level ACDF. Within that group, two patients had 1 level with successful fusion and 1 level with unsuccessful fusion; five patients had successful fusion at both levels. One patient, who was a smoker and underwent a single-level ACDF, had symptomatic pseudoarthrosis that required a revision ACDF. Her revision ACDF took place 13 months following index surgery and it was successful. We performed statistical analyses to identify cellular parameters that could be useful in predicting fusion outcome. For this purpose, the patients were initially categorized based on Lenke’s criteria where a score of 2 or below was defined as successful fusion, but the high fusion rate prevented meaningful comparisons. When clinical outcome was categorized based on a more conservative definition of fusion (defined as patents with a single level score of 1 or two-level score of 1,1 or 1,2), group sizes were comparable (54% fused). We could detect no correlations between fusion outcome and MNC yield, CFU-F yield, or CFU-O measurements (Table 4). However, we detected a strong, yet counterintuitive relationship between CFU-F frequency (CFU/MNC) and fusion (p = 0.0067, Table 4, Fig 3F) in that CFU-F frequencies of less than 5.83x10^-5^ were more likely to be associated with the fusion group. This relationship was not apparent when CFU measurements were normalized to the volume of the original CBMA specimen, suggesting that the ratio of MSCs to other bone marrow cell types was the critical factor responsible for the observations rather than the absolute dose of MSCs in the sample. The data also indicated that tobacco usage was associated with the suboptimal fusion group (p = 0.0402, one tailed Fisher exact test, one direction of interest).

**Table 4:** CBMA parameters and fusion outcome.


Comparison	Mean and SEM	Test	P-value	Notes
**MNC/mL vs fusion**	Yes 4.520x10^-7^± 8.929x10^-6^ No 3.616x10^-7^± 7.628x10^-6^	T-test	0.45	
**CFU-F/mL vs fusion**	Yes 1650 ± 311.6 No 2178 ± 711.1	T-test	0.51	
**CFU-F/MNC vs fusion**	Yes 3.273x10^-5^± 4.375x10^-6^ No 5.961x10^-5^± 8.429x10^-6^	T-test	0.0067	Greater probability of fusion in samples (3.0x10^-6^–5.83x10^-5^ CFU-F/mL) **
**CFU-O/mL vs fusion**	Yes 926.8 ± 215.2 No 815.3 ± 268.4	T-test	0.74	
**CFU-O/MNC vs fusion**	Yes 2.249x10^-5^± 5.541 x10^-6^ No 1.799 x10^-5^± 4.425 x10^-6^	T-test	0.25	

Comparison of CBMA cellular parameters with fusion outcome with fusion defined by Grade 1 according to the modified Lenke’s Criteria (Table 1).

** represents statistical significance (p<0.01).

## Discussion

Although multiple studies support the use of BMA in spinal fusion surgeries [8, 9, 19], limited data are available when it comes to evaluating the relationship between cellular yield and composition of BMA to fusion rates. This is surprising given that overall cell yield and CFU frequency of BMA samples can vary substantially among individuals [20, 21]. Herein, we evaluated the relationship between the cellularity and mesenchymal composition of CBMA to spinal fusion outcome in human patients undergoing ACDF with autologous bone marrow.

While reports vary, it is generally accepted that donor age can adversely affect cell recoveries in BMA, and CFU frequencies are more likely to be adversely affected in females than in males with age [13, 21, 22]. The composition of BMA can also be affected by aspirate volume and it has been reported that large aspiration volumes have the capacity to dilute the sample with blood and thus reduce the effective concentration of CFU-F forming cells [20, 23]. The location of the iliac aspirate has also been reported to affect the frequency of MSCs [24, 25]. In consideration of these sources of variation, our aspiration procedures were normalized in terms of volume and anatomical location. The duration between recovery, processing and implantation can also affect cell yields, [26] but all processing times were minimized and we detected no significant loss in cell viability after processing. We also observed that of the CBMA and BMA samples tested, all generated plastic adherent MSC cultures with the expected immunophenotype and the capacity to form osteoblasts, adipocytes and chondrocytes under standard conditions of differentiation (Fig 2F–2K).

In agreement with previous studies [13, 21, 22], we observed a negative trend between age of donor and MNC recovery when the results were normalized to the volume of the sample (Table 2, Fig 3A). We also observed a similar negative trend with CFU-F yields (Table 2, Fig 3B) when normalized to sample volume but this relationship was lost when normalized to total MNC number, presumably due to the simultaneous reduction in MNCs with age. In the majority of cases, a significant proportion of CFU-F are MSCs and thus have the capacity to differentiate into mineralizing osteoblasts under appropriate conditions (Fig 2C, 2E, 2F and 2H) [27–29]. When this assay was performed on CFU cultures, we found that in females, but not in males, the potential for CFU-F colonies to differentiate into mineralizing osteoblasts (CFU-O) diminishes with age (Table 2, Fig 3C and 3D). These observations agree with Muschler *et al*. [21] who performed similar assays using alkaline phosphatase expression to visualize osteoprogenitors (CFU-AP). In their study, age-related declines in CFU-AP frequency were most evident when genders were separated and the strongest correlation was observed when samples were normalized to sample volume rather than CFU frequency. Hernigou *et al*. [13] reported similar gender-specific results using with a colony visualization technique that did not assay for osteogenic capacity. In our study, we did not observe gender-specific correlations between age and basic CFU-F yields (CFU/mL) or frequency (CFU/mL) (Table 2), but this could be due to our smaller dataset and the background from CFU-Fs that do not exhibit multipotency.

MNC yields were equivalent between males and females, in agreement with previous reports [13, 21, 22] and we did not detect gender-related differences in CFU-O yields or frequencies when all ages were combined (Table 3). Interestingly, we did find that CFU-F frequencies (but not yields) were higher in females than in males (Fig 3E), a result that is not due to MNC values and an observation not shared by a similar study conducted by Hernigou *et al*. [13]. Because the patient demographics and sample recovery procedures were comparable, reasons for these contrasting observations are presently unclear. Nevertheless, it is important to note that colonies were defined differently in the two studies; >3 mm in this study (about 2000 confluent cells, Fig 2D) and >50 cells in Hernigou *et al*. and the durations of the assays were 21 days without media change and 10 days with media changes respectively. While contrasting methodology is an important concern, it is necessary to note that our data could be explained in terms of reported gender-specific differences in cellular physiology. For example, increased stem cell populations in females due to past exposure to pregnancy-related factors have been described [30] and MSCs recovered from female patients undergoing joint surgery have been reported to exhibit increased proliferative potential as compared to male MSCs [31].

Tobacco use is associated with poor healing capacity suggesting that factors in tobacco could directly affect the cellular composition and osteogenic potential of bone marrow. Surprisingly, several studies have reported that tobacco use does not have a detrimental effect on bone marrow cellularity and the formation of CFU-F [22, 32, 33]. Our studies are in agreement with these reports (Table 3).

The fusion rates obtained in our study (86.7%) were similar to previous studies that utilized BMA in conjunction with an osteoconductive agent [8, 19, 34]. Due to the high rate of fusion (as defined by Grade 1 or 2 of the Lenke’s modified criteria), the non-fused group size was not large enough to perform meaningful analyses on the potential influences of CBMA criteria and outcome. We therefore performed a series of tests where fusion was defined by patients with a single level score of 1 or two-level score of 1,1 or 1,2. This categorization, while more conservative, resulted in equivalently sized groups. Even after redefining the groups, we did not detect any relationships between CFU-O potential and fusion outcome, an unexpected result given the postulated role of MSCs in bone maintenance and regeneration. We did, however, make a surprising observation in that a lower ratio of CFU-F to MNCs correlates with an improved incidence of fusion (Fig 3F, Table 4). Specifically, we found that a CFU-F frequency of <5.83x10^-5^ CFU-F/MNC (range: 3.0x10-6–5.83x10^-5^) was more likely to be associated with the fusion group with a robust p-value of 0.0067. In contrast, the yield of CFU-F was not associated with fusion outcome. These results, although initially counter-intuitive suggest that the efficacy of CBMA is dependent on the balance between the number of CFU-F competent cells and other cellular constituents in the bone marrow rather than the absolute dosage of CFU-Fs administered. Furthermore, the lack of correlation between CFU-O and fusion outcome posit that characteristics other than the ability of MSCs to differentiate into osteoblasts play a key role in spinal fusion.

In a study of the effect of CBMA on the healing of tibial non-unions, Hernigou *et al*. reported that the number of CFU-Fs administered and the concentration of CFU-Fs in the graft positively correlated with successful bone healing [13]. At first appraisal, the Hernigou *et al*. data appear to contrast with those reported here, but if one assumes the CFU-F assay protocols are equivalent and calculates the range of CFU-F frequencies from the Hernigou data, they would be predicted to be between 2.5 x 10^−5^ and 4.1 x 10^−5^, generally below the high CFU-F frequencies that predict suboptimal clinical outcome in the work presented here. The collective outcome of both studies therefore suggest that while the Hernigou study identified the minimal effective CFU-F frequencies for bone repair, the results presented herein provide information on the maximal CFU-F frequency.

One explanation for our observations may lie in the potent immune-modulatory capacity of undifferentiated MSCs present in the CFU-F fraction of the CBMA [35–37]. MSCs have been reported to inhibit virtually every facet of the adaptive and innate immune system including the inhibition of inflammatory T-cell activity, activation of regulatory T-cells and the inhibition of inflammatory macrophage activity [38]. Therefore, while they are a necessary source of osteoblast progenitors, concentrated levels of undifferentiated MSCs could inhibit the T-cell and macrophage-mediated inflammatory cascades that are essential for the initial stages of bone repair [39–41].

Another intriguing explanation for our results is prompted by reports that in mice, a population of osteoblasts originate from long-term repopulating hematopoietic stem cells (HSCs) [42]. It is well established that MSCs have the capacity to regulate HSC biology [43] and one would therefore expect that the relative sizes of the mesenchymal and hematopoietic pools in bone marrow could profoundly affect osteogenesis. Our results contribute to this notion, indicating that optimal CFU-F frequency as well as cell dosage should be considered for predicting the outcome of CBMA-mediated ACDF. Interestingly, a study on the effects of CBMA on lumbar fusion reported that outcome trended positively with HSC/mL, but CFU-F frequencies were not examined in detail [4]. In future studies, comparisons of clinical outcome with relative proportions of CFU-Fs to HSCs and other cellular components in CBMA would undoubtedly provide more insight into the mechanism of CBMA-mediated osteogenesis.

The association of tobacco use with poor clinical outcome in orthopedic spine procedures has been documented for several years [44] but negative effects seem to be more evident for lumbar than cervical procedures [45, 46]. While tobacco use did not affect the cellular parameters in our study, it was weakly but significantly associated with a suboptimal fusion outcome.

The results presented herein demonstrate that CBMA and allograft is a safe and highly effective method for ACDF procedures. While most cellular and demographic factors do not affect the efficacy of CBMA, lack of tobacco use and optimal CFU-F frequency (3.0x10^-6^–5.83x10^-5^ CFU-F/MNC) is associated with an optimal clinical outcome. The observation that an optimal range of MSCs in bone marrow (as it relates to total cell number) is correlated with bone healing efficacy rather than simply the magnitude of the dose of MSCs in the marrow is unexpected, but is enlightening with respect to the mechanism of bone healing by complex, minimally manipulated autologous cellular preparations. These data are of substantial and broad relevance when one considers the increased use of variable and uncharacterized autologous bone marrow and lipoaspirate preparations for a range of therapeutic procedures under the loose definition of “*stem cell*” therapies. As opposed to purified stem cell preparations, minimally manipulated tissue isolates contain stem cells, but also countless other cell types that can affect their efficacy in unpredictable ways.

## Conclusions

The objective of this study was to compare patient demographics, bone marrow cellular yields, bone marrow MSC frequencies and MSC osteogenic potential with outcome when CBMA is used to perform ACDF. After 1-year, fusion rates were good (86.7%) with pain and disability significantly improved. There was a negative relationship between MNC and CFU-F measurements with age of patient and CFU-Os negatively correlated with age in females but not males. Tobacco use did not affect cellular parameters of CBMA but was associated with poorer clinical outcome. While high-grade fusion was not associated with CFU-O, it correlated strongly (p<0.0067) with CBMA containing the lowest frequencies of CFU-F (3.0x10^-6^–5.83x10^-5^ CFU-F/MNC). The optimal frequency of CFU-F as it relates to other cells in the CBMA population may affect ACDF outcome, and the homeostatic ratios between mesenchymal progenitors and other cell types in bone marrow appear to contribute profoundly to osteogenic potential.

## Supporting information

S1 Table Raw data. (XLSX)
